# *In silico* identification and *in vitro* assessment of a potential anti-breast cancer activity of antimicrobial peptide retrieved from the ATMP1 *Anabas testudineus* fish peptide

**DOI:** 10.7717/peerj.15651

**Published:** 2023-07-19

**Authors:** Douglas Law, Ahmed Abdulkareem Najm, Jia Xuan Chong, Joelene Zi Ying K’ng, Mas Amran, Huey Lih Ching, Rui Rui Wong, May Ho Leong, Ibrahim Mahmood Mahdi, Shazrul Fazry

**Affiliations:** 1 Faculty of Health and Life Sciences, INTI International University, Nilai, Negeri Sembilan, Malaysia; 2 Department of Food Sciences, Faculty of Science and Technology, Universiti Kebangsaan Malaysia, Bangi, Selangor, Malaysia; 3 Molecular Diagnostic Department, Karl Kolb GmBH & Co, KG, Dreieich, Germany

**Keywords:** *Anabas testudineus*, Antimicrobial peptide, Breast cancer, Peptide drug

## Abstract

A previous study has shown that synthetic antimicrobial peptides (AMPs) derived from *Anabas testudineus* (ATMP1) could *in-vitro* inhibit the progression of breast cancer cell lines. In this study, we are interested in studying altered versions of previous synthetic AMPs to gain some insight into the peptides functions. The AMPs were altered and subjected to bioinformatics prediction using four databases (ADP3, CAMP-R3, AMPfun, and ANTICP) to select the highest anticancer activity. The bioinformatics *in silico* analysis led to the selection of two AMPs, which are ATMP5 (THPPTTTTTTTTTTTYTAAPATTT) and ATMP6 (THPPTTTTTTTTTTTTTAAPARTT). The *in silico* analysis predicted that ATMP5 and ATMP6 have anticancer activity and lead to cell death. The ATMP5 and ATMP6 were submitted to deep learning databases (ToxIBTL and ToxinPred2) to predict the toxicity of the peptides and to (AllerTOP & AllergenFP) check the allergenicity. The results of databases indicated that AMPs are non-toxic to normal human cells and allergic to human immunoglobulin. The bioinformatics findings led to select the highest active peptide ATMP5, which was synthesised and applied for *in-vitro* experiments using cytotoxicity assay MTT Assay, apoptosis detection using the Annexin V FTIC-A assay, and gene expression using Apoptosis PCR Array to evaluate the AMP’s anticancer activity. The antimicrobial activity is approved by the disc diffusion method. The *in-vitro* experiments analysis showed that ATMP5 had the activity to inhibit the growth of the breast cancer cell line (MDA-MB-231) after 48 h and managed to arrest the cell cycle of the MDA-MB-231, apoptosis induction, and overexpression of the p53 by interaction with the related apoptotic genes. This research opened up new opportunities for developing potential and selective anticancer agents relying on antimicrobial peptide properties.

## Introduction

Cancers are a significant public health problem worldwide and are the second leading cause of death globally, accounting for about 9.8 million deaths in 2021 (World Health Organization, 2022). Cancer continues to grow globally, exerting tremendous mental, physical, and financial strain on individuals, communities, and health systems (World Health Organization, 2022). Breast cancer is the most frequent type among women, influencing 2.1 million women each year. More molecular-level research is required to assess breast cancer’s prognosis and clinical management, a paramount public health concern (Feng et al., 2018).

AMPs have recently attracted the interest of several researchers due to their capacity to suppress microorganisms and tumours (Jin & Weinberg, 2019). These peptides are divided into several categories according to their physicochemical characteristics, such as net charge, secondary structure content, and solubility. They are low molecular weight proteins with broad antimicrobial and immunomodulatory activity against infectious bacteria, viruses, and fungi. AMPs have been investigated as a potential cancer therapy technique alone or combined with other traditional medicines (Boparai & Sharma, 2020). Hsu, Li-Chan & Jao (2011) isolated two AMPs from dull fish muscle and then tested them *in vitro* against an MCF-7 human cell line derived from breast cancer patients using papain and proteases. Anchovy fish-derived hydrophobic AMPs increased caspase-3 and caspase-8 activity, leading to apoptosis in human U937 lymphoma cells (Chen, 2016; Lee et al., 2003). Human fibrosarcoma (HT1080 cell line) growth was suppressed in tests using hepcidin TH2-3 from the tilapia (*Oreochromis mossambicus*) fish (Chen, Lin & Lin, 2009). AMPs are expected to target negatively charged cell membrane components, including teichoic acid and lipoteichoic acid, and interfere with intracellular signalling; this improves their effectiveness compared to drugs that only target specific components and specific targets on the cell membrane surface. Since cancer and bacterial cells cannot resist AMPs, they have become an important drug that will significantly change modern antimicrobial use (Najm et al., 2021).

Our previous research on *Anabas testudineus*, a fish commonly found in Malaysia’s freshwater, is a hardy fish that can survive in different environments such as mud and water. One of the main contributions to fish survivability is the epidermal mucus (EM), a shield against environmental biotic and abiotic factors. Our previous research isolated and synthesized two AMP from the skin mucus of *A. testudineus* and examined their effect on breast cancer cell lines (Najm et al., 2022a, 2022b; Alijani Ardeshir et al., 2020). ATMP1 showed significant anticancer activity against breast cancer cell lines (MDA-MB-231 and MCF7) with a nonsignificant effect against skin fibroblast cell line (HS27) (Najm et al., 2021). ATMP1 arrests the breast cancer cell line at the G0/1 phase by interacting and regulating the apoptotic genes (BAX, BCL2, P53, CASPASE3, CASPASE7, and CASPASE8). Thus, this study aimed to develop synthesized antimicrobial peptides with higher anti-breast cancer activity and less toxicity in normal cells by altering ATMP1 using directed evolution theory. We use *in silico* prediction tools to produce peptides with potential anticancer activity and to predict the role of these peptides in inhibiting the growth of cancer cell lines.

The three-dimensional (3D) structure of peptides may be altered and improved to produce antimicrobial peptides (AMPs) with increased efficacy, selectivity, and reduced toxicity. The 3D structure of the peptides was used to create a distinct model for predicting anti-cancer peptides (ACPs), hemolytic peptides, and hazardous peptides (Zhao et al., 2021; Sugrani et al., 2020; E-Kobon et al., 2016; Lamiable et al., 2016). Additionally, the previous study stated that there is a need to develop more peptides and proteins to increase their efficacy and selectivity against cancer cells (Rozek et al., 2000; Chen, Lin & Lin, 2009). Enhancing AMP’s selectivity and cationic properties will minimize its toxicity to eukaryotic cells and increase its potential therapeutic index (Zhao et al., 2021; Thomsen et al., 2020). Therefore, new design approaches are needed to identify more potent sequences (*i.e*., more unmodified sequences without post-translational modifications) that are more effective, have multiple activities without toxicity on normal cells, and have a good selectivity profile. Expanding this field of study is crucial for improving the work described above (Yang et al., 2013; Najm et al., 2022b; Azfaralariff et al., 2022). Toward this end, we aimed to alter ATMP1 and evaluate its cytotoxic activity against cancer cells.

## Materials and Methods

### Antimicrobial peptide design and alteration

Using the directed evolution technique, this study altered single amino acids within the peptide to generate novel peptides. The peptide used in this work was the model peptide ATMP1. Twenty different amino acids are used to alter the peptide (alanine, arginine, asparagine, aspartic acid, cysteine, glutamine, glutamic acid, glycine, histidine, leucine, lysine, methionine, phenylalanine, proline, serine, threonine, tryptophan, tyrosine) in this stud we altered each residue with other amino acids to create new peptide sequences. The list of peptides can be found in Table S1.

### *In-silico* prediction of antimicrobial peptides

**Figure 1:**
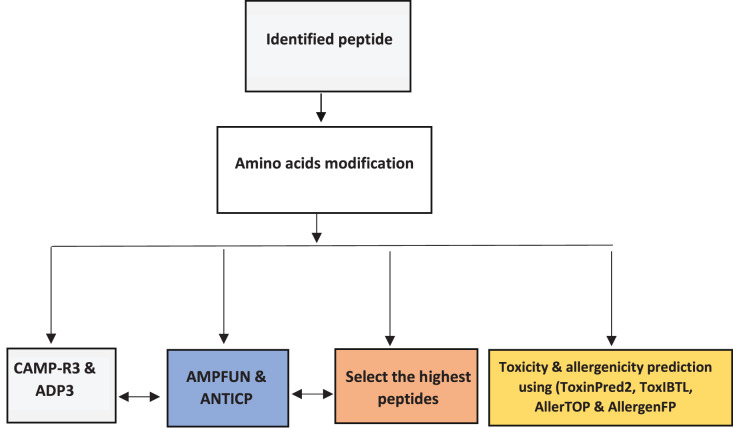
Workflow for bioinformatics prediction and selection of the highest activity of antimicrobial peptides.

The altered peptides’ properties and activities were predicted using AMPs databases (ADP3, CAMP -R3, and AMP fun). Each AMP is submitted individually for AMPs prediction using ADP3 (https://aps.unmc.edu/) and CAMP-R3 (http://www.camp.bicnirrh.res.in/) to predict their properties based on characteristics, preserved structures, and an amino acid profile. Then to predict the potential AMPs with anticancer activity, we used AMPfun (http://fdblab.csie.ncu.edu.tw/AMPfun/index.html) (Fig. 1).

#### Definitions of databases

ADP3 is a database used to predict the physiochemical properties of AMPs based on their amino acid sequences, hydrophobicity, net charge, and molecular weight.

AMPfun is a database that can identify the activities of the AMPs with the assistance of the web server using a two-level setting depending on an algorithm that can locate AMPs and determine their actions (Wang, Li & Wang, 2016). AMPfun can indicate whether an AMPs sequence is an AMP with a specific functional activity (Wang, Li & Wang, 2016). This tool uses the best predictive models for AMPs against cancer.

CAMP-R3 (http://www.camp.bicnirrh.res.in/) is a tool created to broaden and speed up research on AMPs families. It can exploit the relative structural composition of AMPs to find and generate novel AMPs. Besides links to Uniport, PubMed, and other AMPs databases, CAM-PR3 provides details on the sequencing, protein definition, deposit numbers, activity, parent organism, target organisms, and descriptions of specific.

### ANTICP database

To confirm and reliability of Ampfun database prediction, the AMPs were submitted to the ANTICP database. The anticancer activity of every peptide was predicted using the ANTICP online server computer application (https://webs.iiitd.edu.in/raghava/anticp) (Wang, Li & Wang, 2016). To predict and categorize anticancer and non-cancer peptides, SVM algorithms were applied.

### Prot param tool

ProtParam is a program for calculating the physical and chemical properties of proteins. This tool can be found at (http://web.expasy.org/protparam) (Gasteiger et al., 2005). ProtParam was applied to evaluate four properties of two positive peptides (cytotoxic): Instability, PI, hydropathicity, and aliphatic index.

### Prediction of the toxicity of selected peptides

The challenge with protein/peptide therapy is toxicity. This study used two web-based tools, ToxinPred2 and ToxIBTL, applied to predict protein and peptide toxicity. ToxIBTL is freely available at http://server.wei-group.net/ToxIBTL. ToxinPred2 is an *in-silico* method for toxic/non-toxic peptide prediction and design (https://webs.iiitd.edu.in/raghava/toxinpred2/) (Wei et al., 2022; Sharma et al., 2022) As shown in Fig. 1.

### ADME studies

Using the ADMETlab2.0 server, we collected absorption, distribution, metabolism, and excretion (ADME) data for AMPs (Xiong et al., 2021). These studies predicted each peptide and conjugate’s partition coefficient, the permeability of MDCK, and CYP substrate/inhibitor properties.

### Allergenicity prediction

AllerTOP and AllergenFP web servers were utilized to forecast the peptides’ allergenicity. Whereas AllerTOP predicts the allergenicity of peptides using both k-nearest neighbour (kNN) and amino acid E-descriptors, AllergenFP employs five E-descriptor-based fingerprinting (Dimitrov et al., 2014a, 2014b).

### Synthesis of the AMPS

We chose one candidate peptide for *in-vitro* synthesis based on an *in-silico* database’s predictions of peptide activities. We chose only one peptide to synthesise based on these peptides’ anticancer activity and net charge. 1st BASE Co., Ltd. (Singapore) synthesized and supplied the ATMP5 peptide (THPPTTTTTTTTTTTYTAAPATTT).

### Disc diffusion method

This method was applied as previously described by Najm et al. (2021). ATMP5 antibacterial activity was examined against pathogens (*Bacillus subtilis, Bacillus stearothermophilus, Escherichia coli, and Pseudomonas aeruginosa*). The selected strains were spread on the pates of nutrient agar plates and incubated for 24 h at 37 °C. The ATMP5 discs were set by adding 20 μl (200 µg/ml) of ATMP5 diluted in distilled water on a blank antibiotic disc (6 mm). The disc was then put on the nutrient agar plate and incubated for 24 h at 37 °C. The positive control was a conventional antibiotics disc (10 g/ml streptomycin), while the negative control was a blank disc. The inhibition zones surrounding the discs were measured following the incubation to the closest millimetre (mm) (Najm et al., 2021).

### Cytotoxicity effect of crude mucus

#### Cell lines

We purchased the cell lines used in the *in-vitro* study from the American Type Culture Collection Organization. These cell lines include breast cancer (MDA-MB-231) and skin fibroblast cell line (HS27). Table 1 indicates a list of the cell lines used. The culture medium DMEM (Dulbecco’s Modified Eagle Medium), the Trypsin-EDTA solution (0.25%, trypsin activity unit is one mmol/L), the Antibiotic-Antimycotic and Foetal Bovine Serum (FBS) were purchased from Gibco-Thermo Fisher Scientific. The cell lines HS27 and MDA-MB231 were cultured in DMEM, which included 10% heated-inactivate FBS, 100 U/ml penicillin, and 100 g/ml streptomycin. The cell lines were incubated at 37 °C, 5% CO_2_, and 95% humidity.

**Table 1:** Cell lines discerption.

No.	Cell line name	Catalog no.	Description	Type of cell	Passage
1	Hs27	CRL-2496	*Homo sapiens* skin, foreskin	Normal cell line	10–15
2	MDA-MB-231	HTB-26	*Homo sapiens*, human	Cancer cell line	8–17

#### Anticancer activity of peptides

This method was applied as previously described by Najm et al. (2021), Mohammed et al. (2018) using a 3-(4, 5-dimethylthiazol-2-yl)-2, 5-diphenyltetrazolium bromide (MTT) assay. When cell confluence reached 80%, we removed the media from the flask and washed the cells three times in 1X phosphate buffer saline (PBS). After adding trypsin for 5 min to dislodge cells from the flask wall, A hemocytometer was used to count the cell, 1 × 10^5^ of cell concentration seeded into 96 well plates, in each well 200 μl of the suspension.

This extract was added to a well 100 ul for each well plate with different concentrations of ATMP5 (0.625 to 20 μg) and incubated for 24 and 48 h. After 1 day of incubation, 50 mg of the extract was suspended in 1 ml of distilled water. The cytotoxic effect was measured by adding 10 μl of MTT reagent was added to 200 μl of the cell suspension for 4 h. Then measuring absorbance at 570 nm.

#### Annexin V-FITC apoptosis assay

This method was applied as previously described by Najm et al. (2021). The cells were treated with IC_50_ of ATMP5 for 48 h. The cells were stained with 2 µl of Annexin and 2 µl of propidium iodide. BD-FACSCanto II (BD Bioscience, Franklin Lakes, NJ, USA) flow cytometry was used to read and analyse the results.

### RNA isolation and cDNA synthesis

This method was applied as previously described by Najm et al. (2021). Total RNA was extracted from the cancer cell lysate (MDA-MB-231), and the cells were treated with IC_50_ of ATMP5 (7.39 μg/ml) for 48 h. The cell lysate was purified using the AllPrep DNA/RNA Mini Kit (QIAGEN, Kuala Lumpur, Malaysia) following the manufacturer’s guidelines (Ishii et al., 2019). The quality and concentration of the RNA were determined using a spectrophotometer (Thermo Fisher Scientific’s Nanodrop 1000; Thermo Fisher Scientific, Waltham, MA, USA); the absorbance ratio of A260 nm/A280 nm was always >1.9. A total of 20 μl RNA was used as a guide for cDNA synthesis using the RT^2^ First Strand Kit (QIAGEN Company, Kuala Lumpur, Malaysia) according to the manufacturer’s instructions.

To isolate and purify RNA, the genomic DNA was mixed with the reverse transcription mix (20 μl), and the samples were incubated for 15 min at 42 °C followed by 95 °C for 5 min of incubation to stop the reaction (Winkler & McGeer, 2008).

Apoptosis PCR array (96 wells format) was used to carry out pathway-focused gene expression profiling (RT^2^ Profiler PCR array-PAHS-apoptosis, Human PCR array; QIAGEN, Kuala Lumpur, Malaysia) (Cheah & Yamada, 2017). This array allows researchers to test the expression of 84 genes related to human cancer apoptosis and five housekeeping genes. GeneGlobe Data Analysis Centre, a complementary resource for real-time PCR data, analyzed the results.

### Data analysis

Descriptive statistics were applied to the quantitative data in this study. These statistics included standard deviation (SD), average, and percentage. The results were reported as the three independent tests’ standard deviation (SD) and mean (mean). The IC_50_ and mucus components of the extract were also reported using descriptive analysis of percentages. The information was examined using SPSS version 23 and Excel 2016.

## Results

### Peptides alteration

The AMPs’ net charge, total hydrophobicity, and amino acid sequence are all significant factors influencing their bioactivity and toxicity. Efforts to enhance synthetic AMPs frequently seek to alter one or more of these factors, with varying degrees of success (Najm et al., 2022a). In this work, individual amino acids within the peptide were changed to create unique peptides utilising 20 distinct amino acids, with each residue replaced with each of the other 19 amino acids. This improved and increased the previously examined and evaluated anti-cancer efficacy of ATMP1. After altering one amino acid with 20 different amino acids, 428 peptides were identified. These peptides were submitted to the *in-silico* databases to evaluate and select the highest AMPs activity. The complete list of peptides is provided in Table S1.

### AMPs evaluation and selection

ADP3, AMPfun, CAMP -R3, and ANTICP were four bioinformatics programs to predict putative AMPs from peptides. Based on previous studies, the antimicrobial peptides showing significant anti-cancer activity must have properties (a positive net charge, hydrophobicity range (∼ of 15 % or more)), and short-length amino acids of less than 40 amino acids. The preferred net charge of AMPs is from 0 to +2 and amphipathic, which helps the AMPs to interact with and disrupt lipid membranes. Most AMPs are short lengths of fewer than 40 residues (Hoskin & Ramamoorthy, 2008; Sugrani et al., 2020). After screening the results from the four bioinformatics databases, 212 peptides were eliminated as non-antimicrobial peptides, 69 peptides were eliminated as peptides with a negative or deficient net charge, 48 peptides were eliminated as peptides with low hydrophobicity, and the top ten AMPs were selected based on net charge, hydrophobicity, and anticancer activity. As shown in Table 2. ADP3 evaluation of the physicochemical properties of these putative AMPs generated a lower average score for hydrophobicity from 17% to 13% and a significantly higher score for a net charge from 0.0 to +2. Those peptides’ physicochemical properties and the N-terminus, C-terminus, and NC-terminus were then mentioned in the ADP3 and CAMP-R3 datasets. The anticancer activity of the peptides was predicted by the AMPfun database. After this, 10 AMPs were selected as the best peptides with the highest anticancer activity, net charge, and hydrophobicity (as shown in Table 2). Then these peptides were applied to the ANTICP database to confirm the result and to select the top two AMPs with the highest score of anticancer activity. The peptides with less than a 0.5 score were categorized as non-cancer peptides. The ATMP5 (THPPTTTTTTTTTTTYTAAPATTT) and ATMP6 (THPPTTTTTTTTTTTTTAAPARTT) with higher scores of 0.69 and 0.66, respectively, were classified as AMPs with the highest anti-cancer activity. These two peptides were selected for further analysis (as shown in Table 2).

**Table 2:** The 10 peptides shortlisted from the four databases (ADP3, AMPfun, CAMP -R3, and ANTICP) screening.

Peptides name	Sequences	CAMP R3	ADP3		AMPFUN		ANTICP	
			Hydrophobicity	Charge	Activity	Score	Activity	Score
ATMP1	THPPTTTTTTTTTTTTTAAPATTT		12%	0.25	Anticancer	0.0478	AANTICP	-------
ATMP2	THPPTTTTTTTTYTTTTAAPATTT	AMP	13%	0.25	Anticancer	0.3461	ANTICP	0.58
ATMP3	THPPTTTTTTTTTYTTTAAPATTT	AMP	13%	0.25	Anticancer	0.3461	ANTICP	0.57
ATMP4	THPPTTTTTTTTTHTTTAAPATTT	AMP	14%	0.25	Anticancer	0.3403	ANTICP	0.62
ATMP5	THPPTTTTTTTTTTTYTAAPATTT	AMP	18%	1.25	Anticancer	0.3711	ANTICP	0.69
ATMP6	THPPTTTTTTTTTTTTTAAPARTT	AMP	17%	0.25	Anticancer	0.3659	ANTICP	0.66
ATMP7	THPPTTTTTTTTTTHTTAAPATTT	AMP	13%	0.50	Anticancer	0.3403	ANTICP	0.63
ATMP8	THPPTTTTTTTTKTTTTAAPATTT	AMP	13%	0.50	Anticancer	0.3278	ANTICP	0.56
ATMP9	THPPTTTTTTTTTKTTTAAPATTT	AMP	13%	0.50	Anticancer	0.3212	ANTICP	0.55
ATMP10	THPPTTTTTTTTTTTTTAAPATTK	AMP	13%	1.25	Anticancer		ANTICP	0.57
ATMP11	THPPTTTTTTTTTTTTTAAPAKTT	AMP	13%	0.25	Anticancer	0.3253	ANTICP	0.56

### Prediction of the toxicity and allergenicity of selected peptides

ATMP5 and ATMP6, with the highest anticancer activity, were submitted to deep learning databases (ToxIBTL and ToxinPred2) to predict the peptides’ toxicity. The results of both databases predicted that (ATMP5 and ATMP6) AMPs are non-toxic to normal human cells. The results of both databases predicted that ATMP5 and ATMP6 are non-toxic to normal human cells. The ToxIBTL database predicted that ATMP5 and ATMP6 were non-toxic, with scores of 4.1432 and 2.6253, respectively. The Toxinpred2 database also predicted that the ATMP5 and ATMP6 were non-toxic. According to the AllergenFP v1.0 web server, ATMP6 is a potential allergen, whereas ATMP5 is probably non-allergenic. In contrast, the AllerTOP v2.0 web server predicted that the ATMP5 was a probable non-allergen and A was a probable non-allergen (as shown in Table 3).

**Table 3:** Details on bioinformatic prediction of ATMP5 and ATMP6 from TOXIBTL, Toxinpred2, AllerTOP v. 2.0, and AllergenFP v.1.0 databases.

Peptides	TOXIBT *L*		ToxinPred2		AllerTOP v. 2.0	AllergenFP v.1.0
Peptides	Prediction	Score	Prediction	Score	Prediction	Prediction
ATMP5	Non-toxic	4.143273	Non-toxin	−0.73	PROBABLE ALLERGEN	PROBABLE NON-ALLERGEN
ATMP6	Non-toxic	2.625342	Non-toxic	−0.83	PROBABLE ALLERGEN	PROBABLE ALLERGEN

### ADME studies

**Figure 2:**
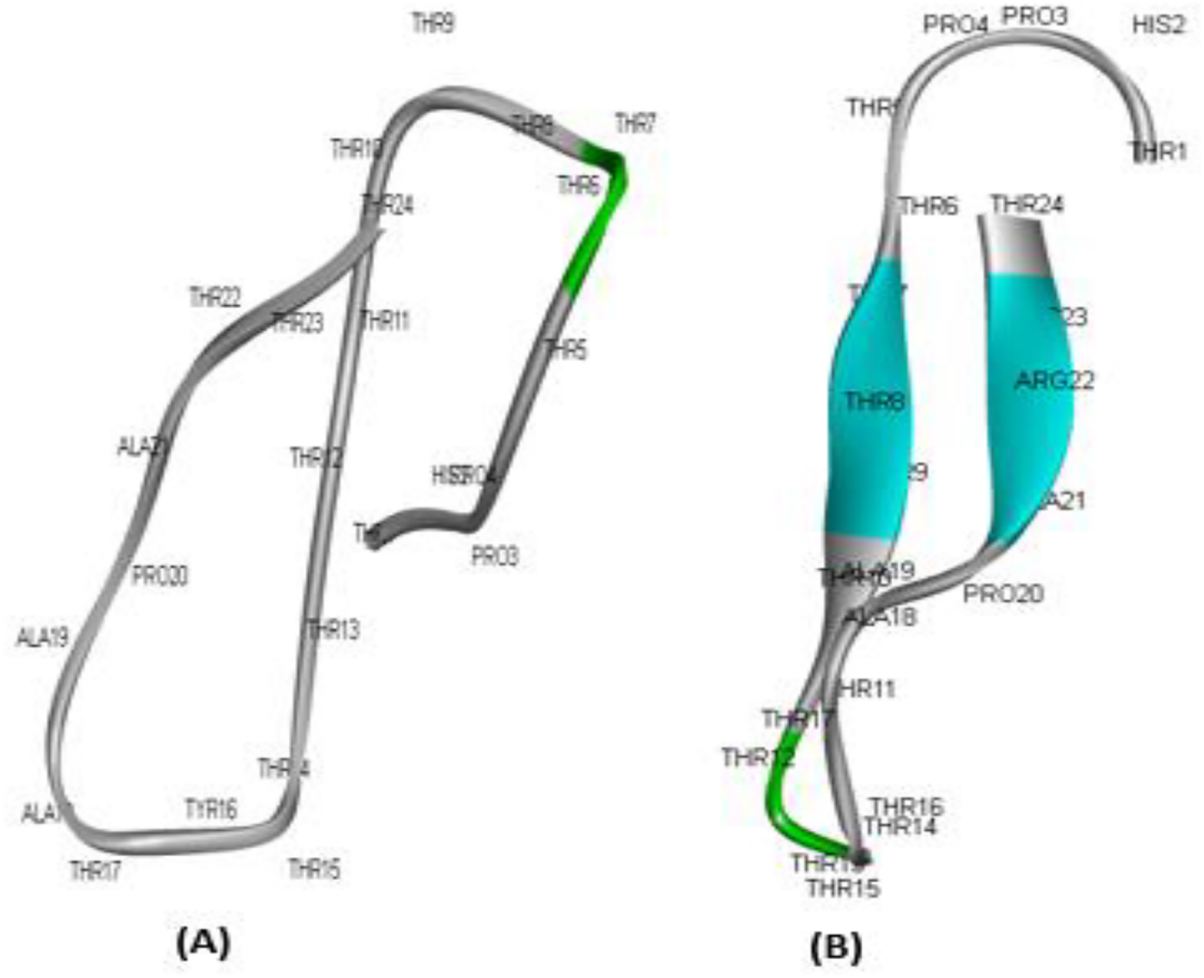
3D model structure for ATMP5 and ATMP6 by using PEPFOLD3. (A) ATMP5, (B) ATMP6.

To determine the potential of the developed compounds as therapeutic candidates, ADME experiments to analyse the pharmacokinetic features of the peptides are crucial (E-Kobon et al., 2016). In particular, *in silico* ADME screening information may be used to pick the most promising compounds and reduce the likelihood that medicine would be rejected (Rozek et al., 2000; Shahid et al., 2021). Consequently, permeability, P-glycoprotein (Pgp) inhibitor/substrate behaviour, CYP enzyme substrate/inhibitor capacity, and hERG blocker capability of Madin-Darby canine kidney cells (MDCK). The MDCK model for permeability has received a lot of attention, and it is commonly believed that its apparent permeability coefficient, Papp, may tell us how well chemicals are taken in by cells (Chen, Lin & Lin, 2009). Additionally, the designed drugs must avoid the blockage of the human ether-a-go-go-related gene (hERG) as it plays an essential role in the interchange of cardiac action potential and resting potential (Wlodkowic, Skommer & Darzynkiewicz, 2009; Kuo et al., 2018). The results are listed in Table 4. The full details of ADME studies are provided in File S1. Both peptides showed MDCK permeability was the highest for ATMP6 (0.001882), In contrast, ATMP5 had a medium permeability of 0.000698. The ATMP5 did not produce CYP substrates or inhibitors, but the ATMP6 did not produce CYP substrates but did produce CYP inhibitors, even though they mostly produced PGP substrates, indicating that this characteristic may impact drug efflux. None of the compounds caused hERG blockage, a desirable drug property. The 3D model structure of the ATMP5 and ATMP6 is shown in Fig. 2.

**Table 4:** ADME studies results.

Compound	MDCK permeability (cm/s)	Herg blocker	Pgp inhibitor/substrate	CYP1A2 substrate/inhibitor
ATMP5	0.000698	No	1.75/1.0	No/No
ATMP6	0.001882	No	0.05/1.0	No/Yes

### Antimicrobial effect of ATMP5

**Figure 3:**
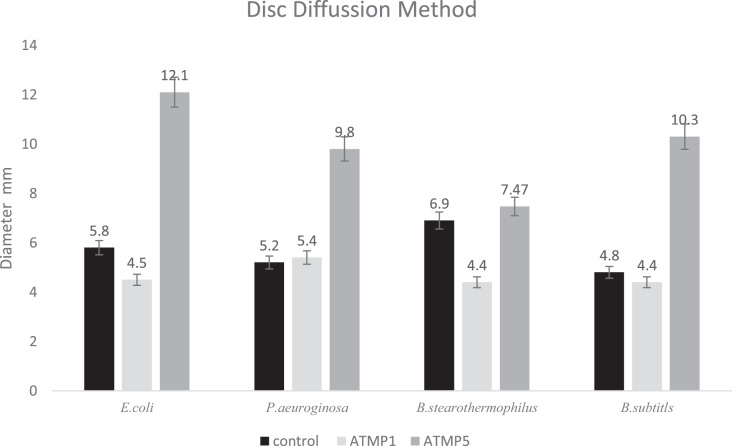
Antibacterial activity of peptides using disk diffusion method. Each disc contains 20 μl (200 µg/ml) of ATMP5. The experiment was repeated with three biological replicates and three technical replicates. The data were expressed as the mean $\pm$ standard deviation (error bar) for three replicates. correlation analysis utilizing the bivariate analysis at a 95% confidence level revealed significant findings (*p* < 0.01).

The antibacterial efficacy of ATMP5 against human pathogens (*E. coli, P. aeruginosa, B. subtilis*, and *B. stearothermophilus*) was evaluated using the disc diffusion technique in comparison to streptomycin as the control. Figure 3 shows the diameter of the inhibition zone that was created. It was discovered that ATMP5 greatly outperforms ATMP1 and the common antibiotic streptomycin in terms of antibacterial activity. To compare the results of ATMP1, ATMP5, and the control, this study utilizes correlation bivariate analysis at a 95% confidence level to reveal significant findings (*p* < 0.01).

### *In-vitro* cytotoxic effect of AMPs

**Figure 4:**
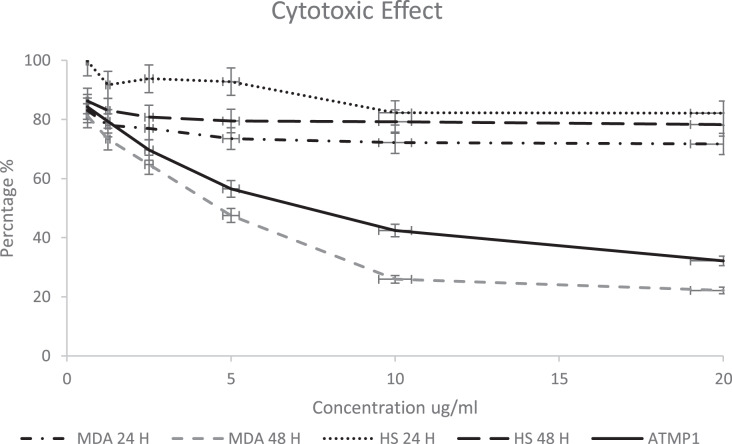
Cytotoxicity effect of synthetic ATMP5 on cancer MDA-MB-231 breast cancer cell line and fibroblast normal cell line (HS27). The cytotoxicity was calculated and the results show the treatment of the cancer cell line after 24 and 48 h. The graphs are representing cell viability (%) by measuring the absorbance of optical density with a microplate reader. The cytotoxic activity was determined by fluorescence measurement using 470 nm excitation/510 nm emission and expressed relative fluorescence units. The values are expressed as mean $\pm$ SEM with standard deviation (standard error of the mean) of three biological and three technical replicates.

The ATMP5 cytotoxicity effect against the human Adenocarcinoma breast Cancer cell line MDA-MB-231 and as a control (human skin fibroblast cell line HS27) was analyzed by MTT assay by treated with the ATMP5 concentration range of 0.625 to 20 μg/ml. It was found that an increase in the concentration of peptides significantly reduced the viability of the MDA-MB-231 cells-line after 24 and 48 h, as shown in Fig. 4. However, ATMP5 didn’t show a significant effect against the HS27 cell line, as shown in Fig. 4. The IC_50_ of the ATMP5 against HS 27 and MDA-MB-231 after 24 h were 96.20 $\pm$ 0.02 μg/ml and 64.04 $\pm$ 0.021 μg /ml respectively; The IC_50_ of the ATMP5 against HS27 and MDA-MB-231 after 48 h was 52.01 $\pm$ 0.14 μg/ml and 7.39 $\pm$ 0.14 μg/ml respectively. The IC_50_ of ATMP1 against MDA-MB-231 after 48 h was 8.25 $\pm$ 0.14 μg/ml (Najm et al., 2021).

### Apoptosis detection results

**Figure 5:**
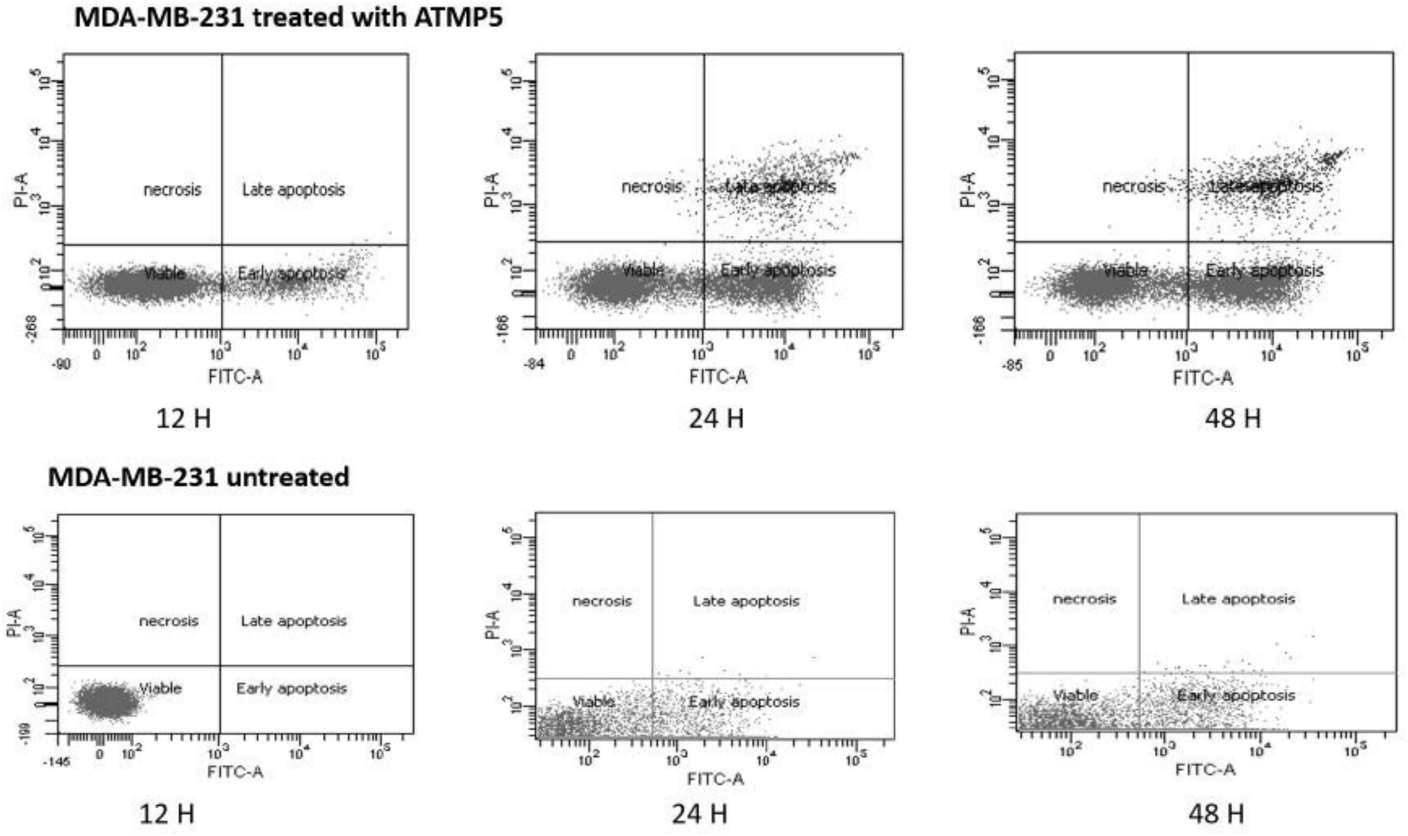
Apoptosis detection results by Anexien V FTIC-A assay. The cells were treated with IC_50_ of ATMP5. This analysis used three-time points 12, 24, and 48 h to detect apoptosis. BD-FACSCanto II (BD Bioscience) flow cytometry was used to read and analyse the results. The values are expressed as mean $\pm$ SEM with standard deviation (standard error of the mean) of three biological and three technical replicates.

The Annexin V-FITC assay was used to find ATMP5 in MDA-MB-231 cell lines that had undergone apoptosis. The impact of ATMP5 MDA-MB-231 was examined in this study using untreated MDA-MB-231 as a control to compare with the treated cell lines. Figure 5 shows the upper right quadrant of necrotic cells, the lower left quadrant of viable cells, the upper right quadrant of late-phase apoptosis, and the lower right quadrant of early-phase apoptosis. These results revealed that the mitochondrial membrane was damaged during the early phases of apoptosis. Phosphatidylserine connected to Annexin V appeared on the cell surface. One of the earliest apoptosis markers is thought to be this occurrence. Meanwhile, late-stage apoptosis lysed the nuclear membrane, allowing the stain to enter the nucleus. As shown in Fig. 5, the early apoptotic cell populations of MDA-MB-231 treated with the ATMP5 at 48 h were about 30.94 $\pm$ 0.12, and the late apoptotic was 12.95 $\pm$ 0.14, respectively. Untreated MDA-MB-231 cells showed about 85% viable cells, which approved our cell cytotoxic effect assay finding. Compared to our previous results, ATMP1 showed that about 25% of the cells were in the early apoptosis phase, while 29% were in the late apoptotic phase (Najm et al., 2021).

### Gene expression

**Figure 6:**
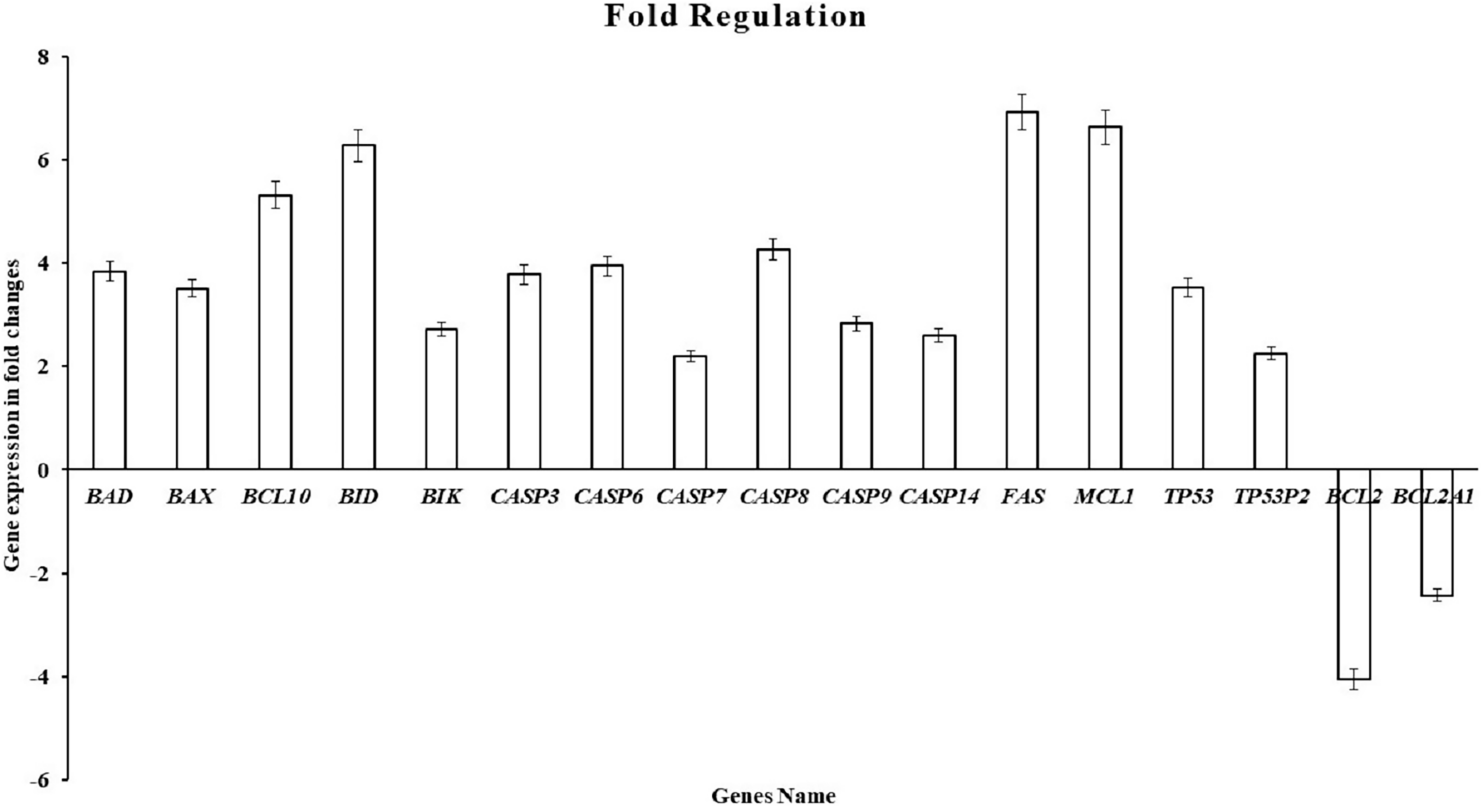
The cell cycle gene expression profile in MDA-MB-231 cells. Apoptosis PCR array (96 wells format) was used to carry out pathway-focused gene expression profiling (RT^2^ Profiler PCR array-PAHS-apoptosis, Human PCR array, QIAGEN, Malaysia). A change of 1.5-fold was used as a selection criterion with the ATMP5 compared with the untreated cells (control). Genes that increased and decreased with at least a two-fold differential expression in test MDA-MB-231cells are represented by the graph. The experiment was repeated with three biological replicates and three technical replicates. The data analysis report was exported from the QIAGEN web portal at GeneGlobe.

By using the human apoptosis cancer RT^2^ Profiler PCR Array (PAHS-012ZA) to detect the gene changes in MDA-MB-231 breast cancer cells. This test included 84 essential gene-regulated human apoptosis. The change in gene expression induced by the ATMP5 in MDA-MB-231 cancer cells at 48 h is summarised in Fig. 6. A change of 1.5-fold was used as a selection criterion with the ATMP5 compared with the untreated cells (control). The details of fold change are summarised in Fig. 5. In this test, many genes were observed to be upregulated or downregulated (details in File S2). However, these genes showed less than a 1.5-fold change; therefore, it should have been addressed. The ATMP5 upregulated the expression of 16 genes in the MDA-MB-231 cancer cell, including BAD, BAX, BIK, BID, BCL10, CASP3, CASP6, CASP7, CASP8, CASP9, CASP14, FAS, MCL1, TP53, and TP53BP2 and downregulated the expression of BCL-2 and BCL2A1.

## Discussion

According to our understanding, the current work is one of the first to look at how cancer and healthy cell lines are affected by antimicrobial peptides produced from *A. testudineus* fish. In order to determine which AMPs have the strongest anticancer action, the current study created novel altered sequences and investigated the influence and role of AMPS in *A. testudineus* mucus on cancer cells using a bioinformatic prediction analysis with four databases (ADP3, CAMP-R3, AMPfun, and ANTICP). The bioinformatics *in silico* research led to the selection of two AMPs, ATMP5 and ATMP6, which showed a high score of anticancer activity (0.69 and 0.66), respectively, which is higher than the ATMP1 score (0.59) (Najm et al., 2021). The other properties which led to select the ATMP5 & ATMP6 were the hydrophobicity and the positive net charge; the net charge for ATMP5 & ATMP6 was (1.25 and 0.25) respectively, while the ATMP1 net charge was 0.25 (Najm et al., 2021). Deep-learning databases were queried to forecast the peptides’ toxicity using the two selected AMPs (ToxIBTL and ToxinPred2). The results of both databases indicated that AMPs are non-toxic to normal human cells. To forecast the allergenicity of the peptides, AllerTOP 2.0 and AllergenFP 1.0 web servers were employed (Dimitrov et al., 2014b; Pierce, Cayan & Thrasher, 2014; Wu, Rice & Wang, 2012). This study offered new opportunities to create highly effective and selective antimicrobial peptide-based cancer treatments.

The results of deep learning databases (ToxIBTL and ToxinPred2) to predict the peptides’ toxicity predicted that both AMPs are non-toxic to normal human cells. The ATMP5 showed the best properties (anticancer score, hydrophobicity, net charge, and ability to interact with apoptotic genes). Thus, the ATMP5 was synthesised and applied for In-vitro assays to approve their cytotoxic effect. The cytotoxic effect of ATMP5 was examined on breast cancer cell line MDA-MB-231 compared to human skin fibroblast (HS27). The MDA-MB-231 cell showed 71% and 22% after 24 and 48 h, respectively; HS27 cell viability was shown at 82% and 72% after 24 and 48 h, respectively. The MDA-MB-231 cell viability showed 71% and 22% after 24 and 48 h, respectively; HS27 cell viability was shown at 82% and 72% after 24 and 48 h, respectively. From this result, this study found that ATMP5 has significant and higher anticancer activity compared to ATMP1, which showed a lower cytotoxic effect against the MDA-MB-231 Cell line (Najm et al., 2021).

An allergenic antigen can cause Th2 cells to become activated, which in turn prompts B cells to produce immunoglobulin E (IgE), which activates eosinophils and causes inflammation and tissue shrinking. The AllergenFP v1.0 web server indicates that ATMP6 is potentially allergenic but ATMP5 is likely not, while the AllerTOP v2.0 web server predicted that the ATMP5 was probable non-allergen and A was probable non-allergen (Wang et al., 2016; Xie et al., 2012; Kroeze & Roth, 2012; Shabestari & Samarghandian, 2013).

The current observation is in line with the findings of Chen, Lin & Lin (2009) discovered that human fibrosarcoma cells might be inhibited by the antimicrobial peptide TH2-3 isolated from the skin mucus of tilapia (HT1080). This current study indicated that ATMP5 could significantly reduce breast cancer cell line MDA-MB-231 viability and cause less toxicity on human skin fibroblast HS27. Furthermore, the ATMP5 managed to arrest the cell cycle of MDA-MB-231 breast cancer cells at the G0/1 phase and induced apoptosis of these cell lines. These results revealed that breast cancer cell line MDA-MB-231, treated with the ATMP5, had cell cycle arrest in the G1 phase, apoptosis induction, and overexpression of the tumor suppressor gene p53. This finding supported the previous study on ATMP1 which led to arresting the MDA-MB-231 at the G0/1 phase and induced apoptosis (Najm et al., 2021; Shi, 2002). The dissipation of the potential mitochondrial membrane and cell death was caused by mitochondrial membrane permeabilization. Antimicrobial peptides may cause a change in mitochondrial membrane permeabilization by inducing cancer cell apoptosis by a mitochondrial membrane rupture. The extrinsic apoptotic pathway can be intersected with the intrinsic apoptotic pathway through caspase-8-activated cleavage of inactive BID (BH3-interacting domain death agonist of the Bcl-2 family of proteins). The release of mitochondrial-membrane-related proteins is then triggered (Li et al., 1999; Alijani Ardeshir et al., 2020). Through BCL-2 inhibition, p53 controls the intrinsic apoptotic pathway by interacting with other genes that regulate mitochondrial permeabilization. Previous evidence also suggested that p53 regulated the transcription of the cyclin-dependent kinase inhibitor (p21), which prompted the G1/S checkpoint arrest. On the other hand, p21 contributes to inhibiting cyclin/CDK complexes, which is an important action in the cell cycle progression (Kroeze & Roth, 2012; Shabestari & Samarghandian, 2013).

## Conclusion

In conclusion, the current study described successfully altering an ATMP1 fish antimicrobial peptide to provide more robust, more durable anticancer action with reduced toxicity against normal human cell lines. This study successfully predicts and synthesis a novel peptide ATMP5 with significant antimicrobial and anti-breast cancer activity with nonsignificant effect on fibroblast normal cells. The ATMP5 showed the highest antibacterial and anticancer properties through *I. silico* prediction (AMPfun and ANTICP). The ATMP5 showed higher antibacterial activities against the human pathogen (*E. coli, P. aeruginosa, B. subtilis*, and *B. stearothermophilus*) compared to the ATMP1 as a positive control. The inhibition zones against *E. coli, P. aeruginosa, B. cereus*, and *B. stearothermophilus* were 12.1 $\pm$ 0.11, 9.8 $\pm$ 0.13, 10.3 $\pm$ 0.32, and 7.4 $\pm$ 0.25 mm respectively (Fig. 3). These values are significantly higher compared to ATMP1 (positive control) against *E-coli* (9.6 $\pm$ 0.12 mm), *P. aeuroginosa* (8.8 $\pm$ 0.20 mm), *B. cereus* (4.4 $\pm$ 0.62 mm) and *B. subtilis* (4.1 $\pm$ 0.15 mm ). The ATMP5 showed the ability to inhibit the growth of gram-positive and gram-negative bacteria (*E. coli*, *P. aeruginosa*, *B. subtilis*, and *B. stearothermophilus*). ATMP5 cytotoxicity effect against the MDA-MB-231 and HS27 It was found that an increase in the peptide concentration significantly reduced the cells’ viability. However, ATMP1 and ATMP5 didn’t show a significant effect against the HS27 cell line (Fig. 4). The IC_50_ of the ATMP5 against HS27 and MDA-MB-231 after 48 h was 52.01 $\pm$ 0.14 μg/ml and 7.39 $\pm$ 0.14 μg/ml respectively while ATMP1 against MDA-MB-231 and HS27 was 59.6 $\pm$ 0.14 and 8.25 $\pm$ 0.14 μg/ml respective.

The result of this study showed that the alteration of single amino acids could produce more potent and efficient novel peptides. ATMP5 antibacterial and anticancer activity compare to ATMP1. According to our knowledge, this is one of the first studies to demonstrate how an antimicrobial peptide isolated from the mucus of the *A. testudineus* fish (ATMP1) can be altered using the directed evolution technique by changing single amino acid to produce more efficient and active peptide (ATMP5).

## Supplemental Information

10.7717/peerj.15651/supp-1 Supplemental Information 1 The full list of peptides.

10.7717/peerj.15651/supp-2 Supplemental Information 2 The full list of gene expression changes.

10.7717/peerj.15651/supp-3 Supplemental Information 3 ADME results.

## Additional Information and Declarations

### Data Availability

The following information was supplied regarding data availability: The raw data is available in the Supplemental Files.
